# Supplementary figures and images for: Identification of key pathways and genes underlying melatonin-enhanced drought tolerance in cotton
Source: PeerJ. 2025 Sep 23;13:e20005. doi: 10.7717/peerj.20005 (PMC12466508; doi:10.7717/peerj.20005)

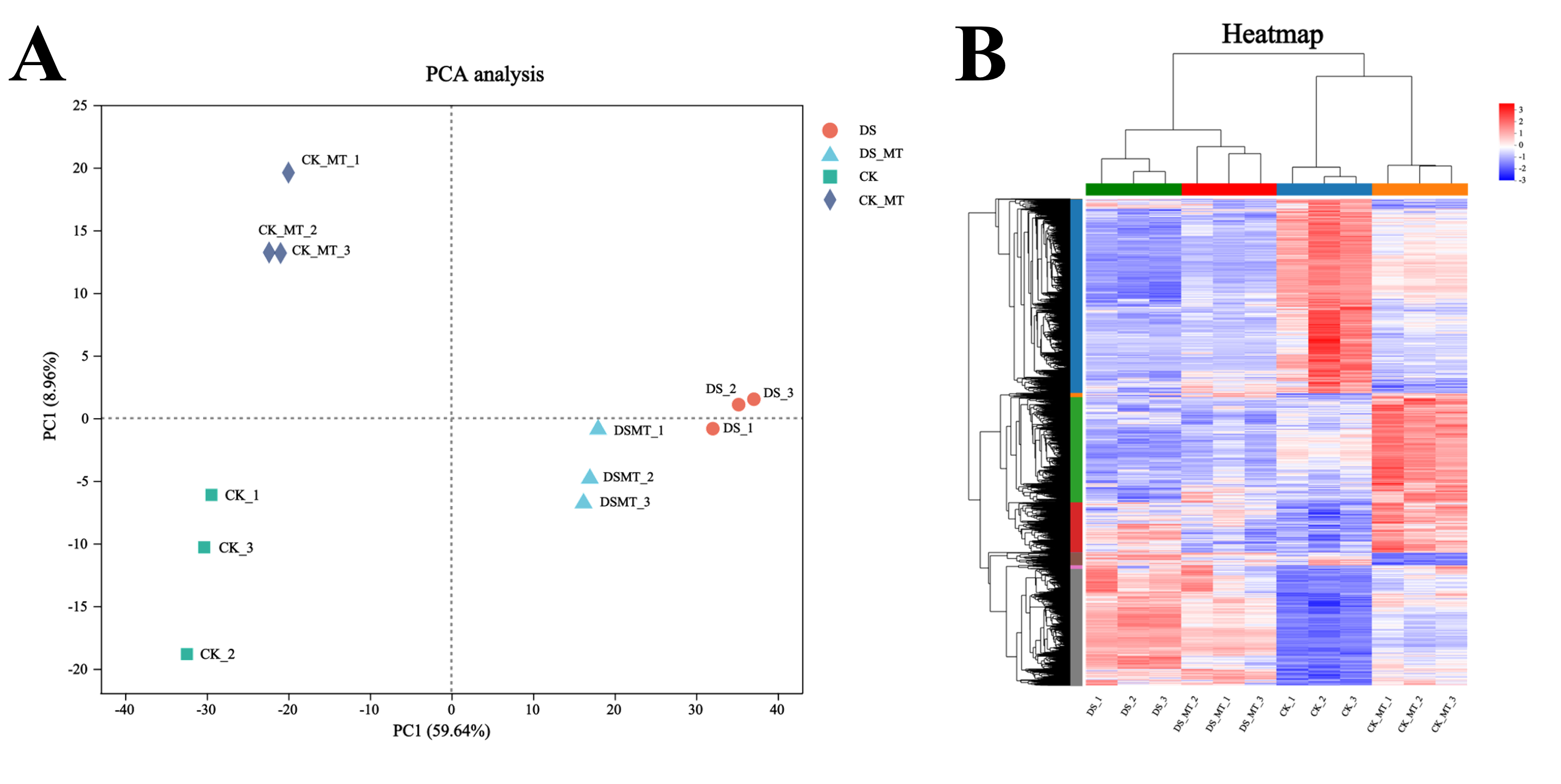

Supplement: Supplemental Information 1 [file peerj-13-20005-s001.png]

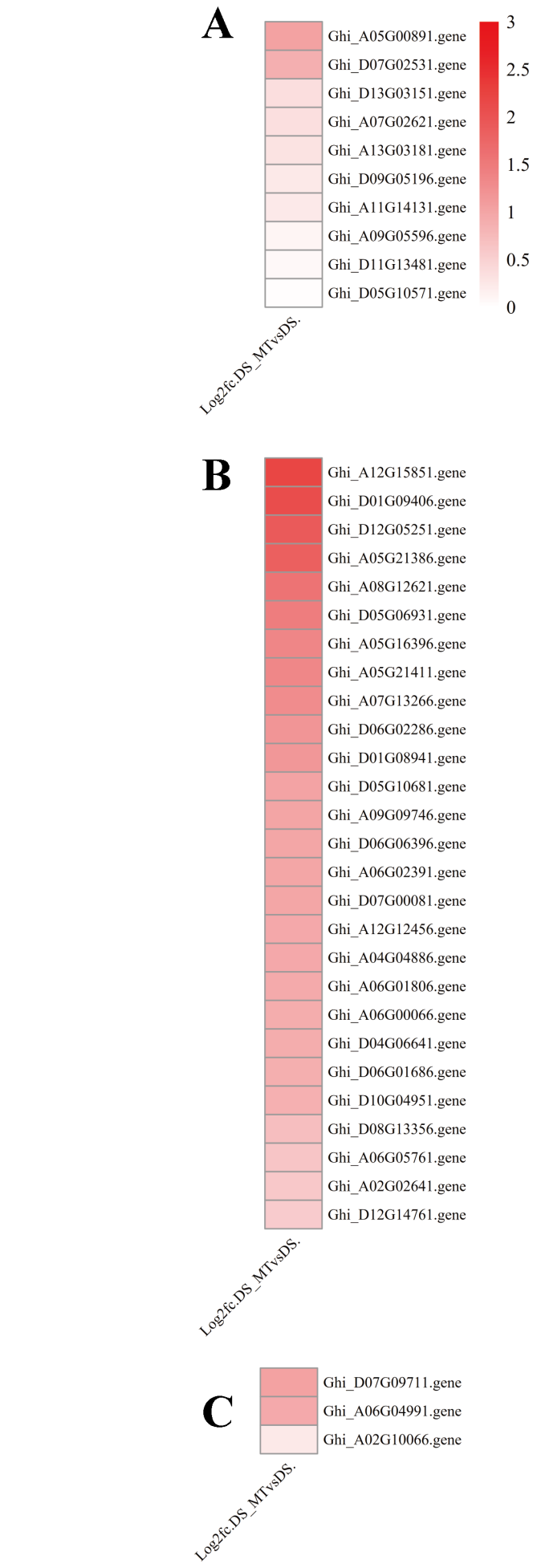

Supplement: Supplemental Information 2 [file peerj-13-20005-s002.png]

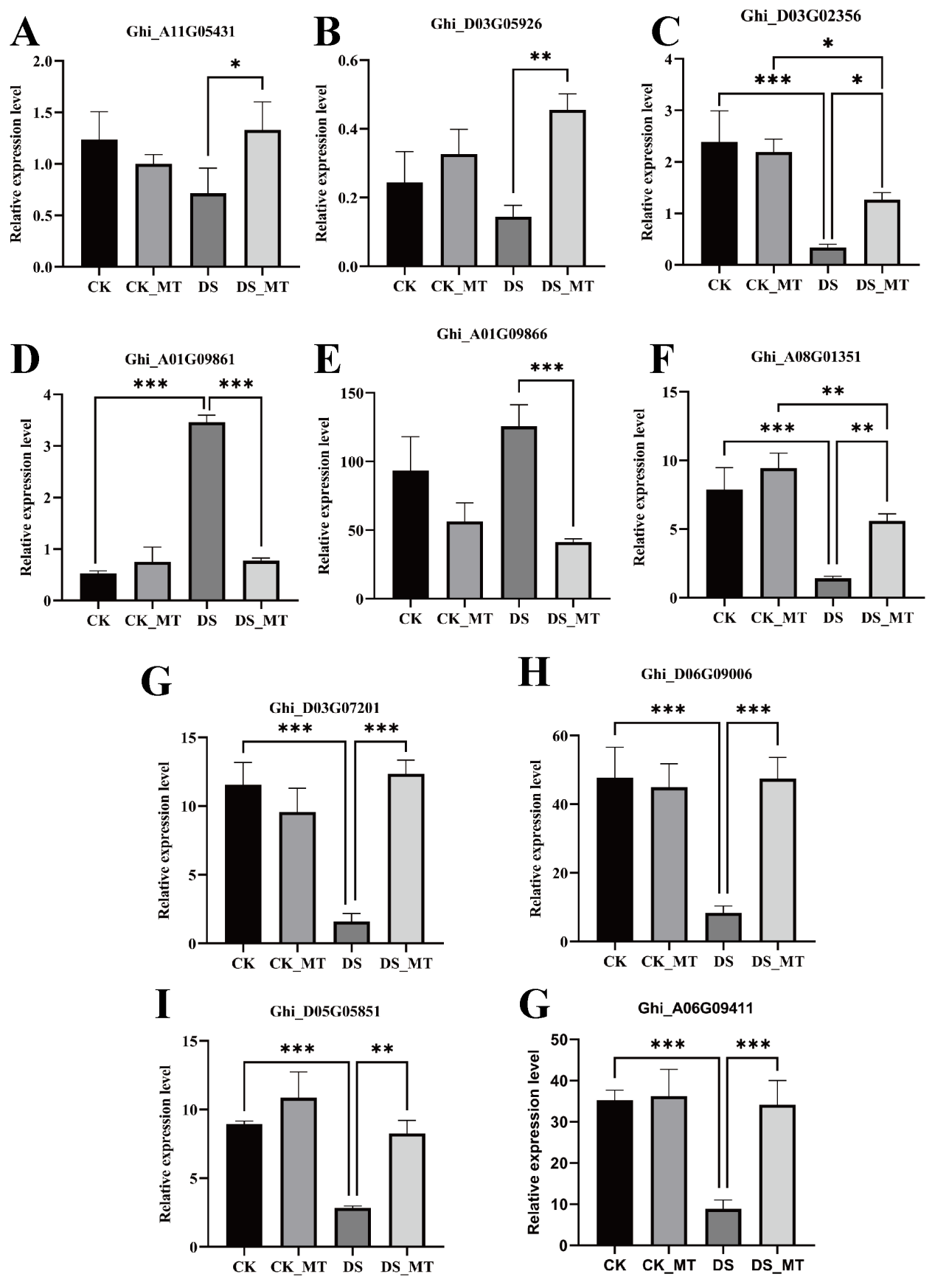

Supplement: Supplemental Information 3 [file peerj-13-20005-s003.png]
