# Supplementary material for: Identification of key pathways and genes underlying melatonin-enhanced drought tolerance in cotton
Source: PeerJ. 2025 Sep 23;13:e20005. doi: 10.7717/peerj.20005 (PMC12466508; doi:10.7717/peerj.20005)
Supplement: Supplemental Information 4 [file peerj-13-20005-s004.docx]

Supplemental Table 1 qRT-PCR primers for 10 DEGs specifically induced by MT under drought **stress.**

| Gene ID | F-Primer | R-Primer |
| --- | --- | --- |
| Ghi_A11G05431 | GTCGTCTGTTCTTGGCTTG | ACAAATCGATGTCATGTTGGTC |
| Ghi_D03G05926 | GCCTTCACACCCAAAACCAC | CGTTTCTTTACCACCGCTGC |
| Ghi_D03G02356 | AATTGTTGCACGTCAAGGCG | TACAACCCCCAAGCAAAGGG |
| Ghi_A01G09861 | ACGCTTTCAACTGAGGTAGGT | GGTATGTTTGGCAGACCACTG |
| Ghi_A01G09866 | GGTATTGAGTATGAAGGTTCCCG | TCATCATCCATGGCTGCATACTC |
| Ghi_A08G01351 | TCAATCGGAGGGTTCAGCAC | GGGGAAGCTTCGTGGTATGT |
| Ghi_D03G07201 | AGGGTCATCCACATGCTCAG | CCCGTAACCATGCTCCTCAG |
| Ghi_D06G09006 | GTCCTCCTCACACAACTCAAAC | TCCACCAGGGCTAACAGATTG |
| Ghi_D05G05851 | AAATCCTCCTCCCAAACCGC | TATGGCGACACGGATGTTGG |
| Ghi_A06G09411 | TCGTCCTCACACAACTCTGC | TAAGCACAAGAGAGGCCAGC |
