# Supplementary material for: Identification of key pathways and genes underlying melatonin-enhanced drought tolerance in cotton
Source: PeerJ. 2025 Sep 23;13:e20005. doi: 10.7717/peerj.20005 (PMC12466508; doi:10.7717/peerj.20005)
Supplement: Supplemental Information 5 [file peerj-13-20005-s005.docx]

| the name of sample | concentration(ng/μl) | total(μg) | OD260/280 | OD260/230 | RQN |
| --- | --- | --- | --- | --- | --- |
| CK_1 | 741.31 | 25.95 | 2.20 | 2.47 | 8.00 |
| CK_2 | 830.71 | 29.08 | 2.19 | 2.44 | 8.40 |
| CK_3 | 557.26 | 19.50 | 2.11 | 2.31 | 9.00 |
| CK_MT_1 | 678.12 | 23.73 | 2.18 | 2.51 | 7.60 |
| CK_MT_2 | 423.81 | 14.83 | 2.16 | 2.30 | 7.40 |
| CK_MT_3 | 818.32 | 28.64 | 2.16 | 2.52 | 7.50 |
| DS_1 | 651.33 | 22.80 | 2.19 | 2.51 | 7.00 |
| DS_2 | 481.80 | 16.86 | 2.11 | 2.35 | 7.20 |
| DS_3 | 759.24 | 26.57 | 2.16 | 2.51 | 8.40 |
| DS_MT_1 | 468.86 | 16.41 | 2.14 | 2.33 | 8.00 |
| DS_MT_2 | 619.17 | 21.67 | 2.07 | 2.27 | 8.10 |
| DS_MT_3 | 690.76 | 24.18 | 2.20 | 2.43 | 8.20 |

Supplemental Table 2 RNA Quality of Cotton (*Gossypium hirsutum*) Seedling Leaf
