# Supplementary material for: Identification of key pathways and genes underlying melatonin-enhanced drought tolerance in cotton
Source: PeerJ. 2025 Sep 23;13:e20005. doi: 10.7717/peerj.20005 (PMC12466508; doi:10.7717/peerj.20005)
Supplement: Supplemental Information 6 [file peerj-13-20005-s006.docx]

Supplemental Table 3  Transcriptome Sequencing Data Quality Assessment and GC Content Analysis.

| Sample | Raw reads | Clean reads | Clean bases | Error rate(%) | Q20  (%) | Q30  (%) | GC content(%) |
| --- | --- | --- | --- | --- | --- | --- | --- |
| CK_1 | 43967506 | 43620396 | 6543115137 | 0.01 | 98.42 | 94.89 | 44.44 |
| CK_2 | 43339270 | 43039340 | 6448636017 | 0.01 | 98.48 | 95.03 | 44.31 |
| CK_3 | 44302292 | 43997992 | 6597389839 | 0.01 | 98.48 | 95.06 | 44.23 |
| CK_MT_1 | 41622688 | 41331492 | 6200298844 | 0.01 | 98.44 | 94.93 | 43.98 |
| CK_MT_2 | 42417122 | 42136150 | 6323179554 | 0.01 | 98.48 | 95.06 | 44.05 |
| CK_MT_3 | 43648998 | 43329096 | 6502829930 | 0.01 | 98.47 | 95.04 | 44.16 |
| DS_1 | 43396902 | 43097052 | 6452486728 | 0.01 | 98.50 | 95.13 | 43.59 |
| DS_2 | 42272388 | 42005424 | 6304448936 | 0.01 | 98.50 | 95.12 | 43.58 |
| DS_3 | 40346030 | 40066748 | 6002710054 | 0.01 | 98.43 | 94.90 | 43.52 |
| DS_MT_1 | 43120946 | 42828656 | 6426148844 | 0.01 | 98.50 | 95.10 | 43.69 |
| DS_MT_2 | 44406056 | 44083934 | 6600189975 | 0.01 | 98.51 | 95.19 | 43.88 |
| DS_MT_3 | 43705558 | 43394102 | 6495074165 | 0.01 | 98.49 | 95.10 | 43.97 |
