# Supplementary material for: Identification of key pathways and genes underlying melatonin-enhanced drought tolerance in cotton
Source: PeerJ. 2025 Sep 23;13:e20005. doi: 10.7717/peerj.20005 (PMC12466508; doi:10.7717/peerj.20005)
Supplement: Supplemental Information 7 [file peerj-13-20005-s007.docx]

Supplemental Table 4 Transcriptome data of 10 DEGs specifically induced by MT under drought stress

| Gene ID | Symbol | Gene Description | CK tpm | DS tpm | CK_MT tpm | DS_MTtpm | CK_MT vs CK | DS_MT vs CK_MT | DS vs CK | DS_MT vs DS |
| --- | --- | --- | --- | --- | --- | --- | --- | --- | --- | --- |
| Ghi_A11G05431.gene | MYC5 | Myc-type, basic helix-loop-helix (bHLH) domain | 4.16 | 2.42 | 7.05 | 4.87 | no\|up | no\|down | no\|down | yes\|up |
| Ghi_D03G05926.gene | PIF8 | Myc-type, basic helix-loop-helix (bHLH) domain | 3.55 | 1.99 | 6.98 | 5.13 | no\|up | no\|down | no\|down | yes\|up |
| Ghi_D03G02356.gene | GGT | Gamma-glutamyltranspeptidase | 4.26 | 0.51 | 3.89 | 1.32 | no\|down | yes\|down | yes\|down | yes\|up |
| Ghi_A01G09861.gene | FMO | Flavin monooxygenase FMO | 0.01 | 0.29 | 0.00 | 0.01 | no\|down | no\|up | yes\|up | yes\|down |
| Ghi_A01G09866.gene | FMO | Flavin monooxygenase FMO | 0.06 | 0.40 | 0.05 | 0.03 | no\|down | no\|down | no\|up | yes\|down |
| Ghi_A08G01351.gene | DXS | Glycoside hydrolase family 16 | 1.89 | 0.44 | 4.87 | 1.58 | no\|up | yes\|down | yes\|down | yes\|up |
| Ghi_D03G07201.gene | ThiC | Phosphomethylpyrimidine synthase ThiC | 237.51 | 123.53 | 169.76 | 242.97 | no\|down | no\|up | yes\|down | yes\|up |
| Ghi_D06G09006.gene | Thi4 | Thiazole biosynthetic enzyme Thi4 family | 1092.65 | 289.32 | 849.55 | 713.83 | no\|down | no\|down | yes\|down | yes\|up |
| Ghi_D05G05851.gene | Thi4 | Thiazole biosynthetic enzyme Thi4 family | 525.24 | 158.64 | 385.30 | 414.63 | no\|down | no\|up | yes\|down | yes\|up |
| Ghi_A06G09411.gene | Thi4 | Thiazole biosynthetic enzyme Thi4 family | 1259.28 | 262.59 | 1018.48 | 850.52 | no\|down | no\|down | yes\|down | yes\|up |
