# Supplementary material for: Identification of key pathways and genes underlying melatonin-enhanced drought tolerance in cotton
Source: PeerJ. 2025 Sep 23;13:e20005. doi: 10.7717/peerj.20005 (PMC12466508; doi:10.7717/peerj.20005)
Supplement: Supplemental Information 8 [file peerj-13-20005-s008.docx]

Supplemental Table 5 12 Genes Specifically Induced by MT and Their Functional Annotations Under Drought Stress

| Gene ID | kME | Symbol | Gene Description |
| --- | --- | --- | --- |
| Ghi_A04G05786.gene | 0.74 | AUX1、 WAV5、PIR1， MAP1 | Amino acid transporter, transmembrane domain |
| Ghi_A11G05431.gene | 0.73 | NIG1 、MYC5、BHLH28 | Myc-type, basic helix-loop-helix (bHLH) domain |
| Ghi_D11G16236.gene | 0.71 | Ribonuclease H-like superfamily protein | Ribonuclease H domain |
| Ghi_D03G05926.gene | 0.69 | UNE10、PIF8 | Myc-type, basic helix-loop-helix (bHLH) domain |
| Ghi_A01G01881.gene | 0.68 | NB-ARC domain-containing disease resistance protein | Leucine-rich repeat |
| Ghi_D09G08051.gene | 0.67 | HMP20 | Heavy metal-associated domain, HMA |
| Ghi_A01G02716.gene | 0.66 | RLK4 | Protein kinase domain |
| Ghi_A01G04151.gene | 0.66 | KCO1、TPK1 | Two pore domain potassium channel |
| Ghi_D01G01566.gene | 0.66 | GOX2 | FMN-dependent dehydrogenase |
| Ghi_D01G09501.gene | 0.63 | PMDH2 | Lactate/malate dehydrogenase, N-terminal |
| Ghi_A04G04421.gene | 0.62 | GIM2 、 GAS2 | Oxoglutarate/iron-dependent dioxygenase |
| Ghi_A10G13731.gene | 0.61 | RCA | ATPase, AAA-type, core |
